# Vortical Structures Promote Atheroprotective Wall Shear Stress Distributions in a Carotid Artery Bifurcation Model

**DOI:** 10.3390/bioengineering10091036

**Published:** 2023-09-03

**Authors:** Nora C. Wild, Kartik V. Bulusu, Michael W. Plesniak

**Affiliations:** 1 Department of Mechanical and Aerospace Engineering, The George Washington University, 800 22nd Street NW, Science & Engineering Hall, Suite 3000, Washington, DC 20052, USA; caroline_zalud@gwu.edu (N.C.W.); bulusu@gwu.edu (K.V.B.); 2 Department of Biomedical Engineering, The George Washington University, 800 22nd Street NW, Science & Engineering Hall, Suite 3000, Washington, DC 20052, USA

**Keywords:** physiological pulsatile flow, vortical structures, wall shear stresses, healthy and pre-disposed geometry, cardiovascular disease

## Abstract

Carotid artery diseases, such as atherosclerosis, are a major cause of death in the United States. Wall shear stresses are known to prompt plaque formation, but there is limited understanding of the complex flow structures underlying these stresses and how they differ in a pre-disposed high-risk patient cohort. A ‘healthy’ and a novel ‘pre-disposed’ carotid artery bifurcation model was determined based on patient-averaged clinical data, where the ‘pre-disposed’ model represents a pathological anatomy. Computational fluid dynamic simulations were performed using a physiological flow based on healthy human subjects. A main hairpin vortical structure in the internal carotid artery sinus was observed, which locally increased instantaneous wall shear stress. In the pre-disposed geometry, this vortical structure starts at an earlier instance in the cardiac flow cycle and persists over a much shorter period, where the second half of the cardiac cycle is dominated by perturbed secondary flow structures and vortices. This coincides with weaker favorable axial pressure gradient peaks over the sinus for the ‘pre-disposed’ geometry. The findings reveal a strong correlation between vortical structures and wall shear stress and imply that an intact internal carotid artery sinus hairpin vortical structure has a physiologically beneficial role by increasing local wall shear stresses. The deterioration of this beneficial vortical structure is expected to play a significant role in atherosclerotic plaque formation.

## 1. Introduction

In the United States, about 50% of deaths are caused by cardiovascular diseases such as strokes and heart attacks [1]. Strokes alone affect over 15 million people every year [2], and up to 30% of strokes are estimated to be caused by carotid artery disease [2,3]. Carotid artery disease is often located at the carotid artery bifurcation (CAB), which is situated in the neck. The common carotid artery (CCA) branches off from the aorta and in turn branches into the internal (ICA) and external (ECA) carotid arteries, where the CAB is the bifurcation from CCA into ICA and ECA. The ICA and ECA transport blood to the brain and face, respectively [1].

Carotid artery disease often involves the formation of atherosclerotic plaques. Plaque growth and/or rupture can block blood supply to the downstream brain vasculature. Atherosclerosis describes the local deposit of cholesterol and lipids on the arterial wall and mainly occurs at locations of complex vessel geometry, such as bifurcations. It mostly affects large- and medium-sized blood vessels [1,2,3,4,5,6]. The formation of a stenosis, which requires the implantation of a stent, is a common consequence of atherosclerotic plaque formation, which poses an additional risk for thrombus formation [7].

The innermost layer in the vessel lumen, the endothelium, is exposed to wall shear stresses, which are present in the entire cardiovascular system [2,3]. These shear stresses on the endothelial cells are caused by blood flow over the vessel lumen; they are required to sustain healthy endothelial cells [8]. Atherosclerosis is caused when pathological flow and consequently abnormal shear stresses are present, when they vary in magnitude or have strong temporal variations [1,9]. These pathological mechanical forces are sensed and transmitted by the endothelial cells, resulting in a diseased cell through a mechanotransduction process [10,11]. In atherosclerotic regions, the flow is disturbed, the shear stress is lower than normal [1,12], and the spatial shear stress gradients are large [13,14,15,16]. Due to their crucial role in the origin of cardiovascular diseases, shear stresses triggering plaque formation in the carotid artery bifurcation are of great clinical interest and are investigated using computational fluid dynamic (CFD) simulations.

The CFD studies of patient-specific artery geometries [17], as well as simplified artery models, are both commonly reported in the literature, incorporating 2D [18] and 3D [19,20,21,22] blood vessel geometries. The use of simplified and patient-averaged models allows for a broader understanding of general flow phenomena in the carotid artery bifurcation and their influence on atherosclerosis, and thus to decouple it from specific patient anatomies [23]. In addition to geometric simplification, a steady flow assumption is used broadly [24,25,26]. More realistically, physiological pulsatile flow can be applied by using a pulsatile inflow waveform at the CCA, which is mostly performed in combination with patient-specific geometries [27]. Typically, ‘healthy’ vessel geometries are modeled [28] that are based on general patient averages without any emphasis on the patient cohorts’ actual risk for future plaque development. Atherosclerotic vessel geometries are typically modeled under diseased conditions [17,29], incorporating a local narrowing of the vessels’ diameter in the form of a plaque built-up (stenosis). This paper takes a novel approach by emphasizing a patient cohort’s predisposition toward plaque formation by modeling a nondiseased pre-disposed CAB. We are unaware of any published study investigating flow fields and vortical structures and their impact on the wall shear stress distribution in carotid artery bifurcation geometries with a different predisposition toward the formation of atherosclerotic plaques.

Flow separation in the ICA sinus is observed and correlated to increased atherosclerosis risk [1,19,20,21,22,26,30,31]. Thus, it is hypothesized that the sinus geometry is an important indicator of risk. This includes the bifurcation angle, as a larger bifurcation angle causes larger areas of nonaxial shear stress at the branches’ outer walls [23], and an asymmetric bifurcation is linked to increased atherosclerosis risk [32]. Instantaneous, as well as time-averaged, wall shear stresses are frequently used metrics in determining proatherogenic regions within the geometry [17,19,20,21,22,23,24,27,29,30].

Shear stress as a mechanical stimulation of atherosclerosis is widely studied. There are, however, only a few studies of the complex flow within the carotid artery bifurcation affecting these shear stresses. Thus, our study aims to increase the understanding of the complex flow, including secondary flow and vortical structures, within the CAB and its impact on the wall shear stress distribution. Such an understanding is necessary for the early detection of atherosclerosis by allowing for the identification of patients with pre-disposed increased risk before the onset of the disease and symptoms.

**Figure 1:**
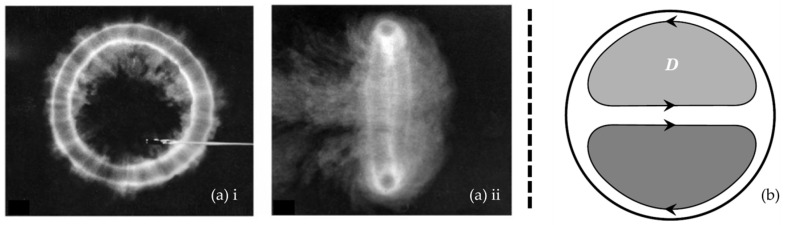
Examples of vortical structures: (**a**) vortex ring front (**i**) and side (**ii**) view [33]; (**b**) schematic of a Dean (D) vortex pair viewed from upstream in a curved pipe; clockwise and counter-clockwise rotation of the secondary flow is indicated by the arrows [34].

A vortex is defined as a specific region of fluid circulating around an axis. The fluid velocity is typically greatest at this axis and decreases with increasing distance to this axis. The vortex core is furthermore a local pressure minimum. A common example of a vortical structure is a vortex ring (Figure 1a). Secondary flows are flows with flow direction in the plane perpendicular to the main flow direction. Those in-plane secondary velocities can develop in pipe flow (similar to a circular cross-sectional vessel) when the pipe or vessel has a curvature. These secondary flows result in Dean vortices, which are a pair of counter-rotating vortices (Figure 1b).

Secondary flows emerge in branching vessels because the axial main flow has to follow two curved paths. The formation of secondary flow structures leads to the formation of vortices, which alter the wall shear stress distribution [35,36,37], its magnitude, and multidirectionality [38,39,40]. Thus, the characteristics of these vortices and secondary flow patterns play a crucial role in the onset of cardiovascular diseases [41]. Very few studies investigate vortices in the flow [17] despite their significant influence on the flow and shear stresses. Rindt et al. found that, at the inlet of the ICA, reversed flows have the same magnitude as the mainstream velocity [18]. Gijsen et al. observed the formation of Dean vortices after the bifurcation under steady flow [26]. These vortices originate from the curvature due to the transition from CCA into ICA, causing the fluid close to the branching dividing wall to be transported toward the nondivider wall. In addition to the increase in cross-sectional area in the ICA sinus, this leads to flow reversal close to the nondivider ICA wall [26]. Kumar et al. reported the helical structure of velocity streamlines downstream of the bifurcation [31]. Morbiducci et al. investigated vorticity in 2D planes in healthy patient-specific geometries under pulsatile flow [21], like Perktold et al. describing the formation of secondary flow structures in 2D planes for a healthy carotid artery bifurcation model under pulsatile flow [30]. The main limitation of these previous studies is the 2D analysis of secondary flows without detailed description or 3D analysis of vortical structures. Nagargoje and Gupta [25] performed a pulsatile computational study in a very simplified carotid artery bifurcation model and found that secondary flows are dependent on sinus size and that a larger sinus increases the low WSS recirculation area, without further specifying secondary flow structures or their 3D structure. One of the few studies to examine 3D vortical structures in a carotid artery bifurcation was published by Chen et al. in 2020 [42]. They investigated patient-specific geometries, with unspecified atherosclerosis risk, finding differences in the time duration of dominant reverse flow and the duration of dominant secondary flows based on varying carotid artery bifurcation geometries. They found that secondary flows are dominant in the deceleration phase at end-systole and that a larger ICA bifurcation angle results in stronger (having higher maximum magnitude) secondary flow during the inflow acceleration throughout the pulsatile cardiac cycle and found the formation of Dean vortices at the systolic peak for geometries having an ICA bifurcation angle around 30°. Complex bifurcation geometry according to Chen et al. breaks the symmetry of the Dean vortices. Furthermore, they observed the formation of straight vortex tubes at the systolic peak. They found that vessels having a higher flare $\left(A_{max}/A_{CCA}\right)$ have larger regions of reversed flow and are more dominated by secondary flows.

Despite the wide range of studies of flow and wall shear stress distribution in carotid artery bifurcation geometries, there is a need for a greater understanding of the evolution and characterization of three-dimensional vortical structures and their role in vascular flow and wall shear stress distribution. The few studies of three-dimensional vortical structures in cardiovascular flows, specifically CAB flow, use them to visualize the three-dimensional distribution of vorticity and helicity. The study by Chen et al. uses classical three-dimensional vortex criteria to highlight secondary flows and flow separation zones. Our study aims to address secondary flow by characterizing vorticial structures using established vortex identification methods in a carotid artery bifurcation model under physiological pulsatile flow. Our objective is to increase the understanding of physiological flow patterns, specifically the role of vortices, in the human carotid artery. The modeling of a representative ‘pre-disposed’ CAB geometry and its hemodynamics using advanced CFD simulations aims to identify pathological flow and vortex patterns. Thus, this study aims not only to increase the understanding of the role of vortices in disease development but also intends to inform the early detection of disease with the focus on identifying patients at high risk that are pre-disposed and likely to form an atherosclerotic plaque—before the plaque develops. Modern biomedical imaging techniques, such as ultrasonography [43] or 4D flow MRI [44,45], allow for the precise visualization of CAB flow in patients, and more emerging techniques such as vector flow imaging allow for the intuitive and quantitative imaging of vortical structures [46]. Helical flow patterns in arteries and vortical flow structures, such as vortex rings in the heart [45], are identifiable in the state of the art of medical imaging and are therefore accessible to clinicians [47]. The clinical analysis of vortices, such as the left-ventricle vortex ring, is often performed by acquiring the flow field using 4D flow MRI [47], extracting vortical structures using the $\lambda_{2}$ criterion [44], and finding its vortex core position [45]. The altered development of a main vortex is associated with pathological cardiovascular flow and function in other parts of the cardiovascular system, such as the diastolic vortex formation in the left ventricle of the human heart [45]. Thus, there is a high potential to transfer this vortex-based clinical evaluation to carotid bifurcation atherosclerosis risk assessment. To achieve this goal, this study focuses on the identification of coherent vortical structure in the CAB and vortex characteristics to distinguish between a physiological or a pathological vortical structure.

There is a lack of knowledge on the impact of three-dimensional vortices in the CAB on possibly proatherogenic shear stress distributions. In order to fill this knowledge gap, we investigate three-dimensional vortical structures in the ICA sinus and their influence on wall shear stress for a representative ‘healthy’ and a proatherogenic ‘pre-disposed’ vessel geometry. This will facilitate a fundamental understanding of vortex evolution over the cardiac cycle and allow for the characterization of the differences between physiological and pathological vortical structures. The accessibility of vortex visualization using medical imaging presents a promising tool to identify high-risk CAB atherosclerosis patients through pathological vortex formation in the future. It can be hypothesized that specific geometrical features affect the vortical structures and thus result in a proatherogenic shear stress distribution. Hemodynamics in a ‘healthy’ and ‘pre-disposed’ CAB geometry are calculated using a three-dimensional CFD simulation with physiological pulsatile flow.

This study will highlight the potential role of three-dimensional vortical structures in atherosclerosis by linking internal vortical structures to the wall shear stress distribution known to critically contribute to disease onset. Furthermore, the results will correlate a statistically ‘pre-disposed’ carotid bifurcation geometry to differing vortex characteristics, aiming to inform the future clinical early detection of CAB atherosclerosis.

## 2. Materials and Methods

The implemented methods will be discussed in the following sections. First, the modeling of statistically ‘healthy’ and ‘pre-disposed’ carotid artery bifurcation geometries is explicated. Second, the three-dimensional computational fluid dynamic simulation and the associated physiological boundary conditions are described.

### 2.1. Carotid Artery Bifurcation Geometry

**Figure 2:**
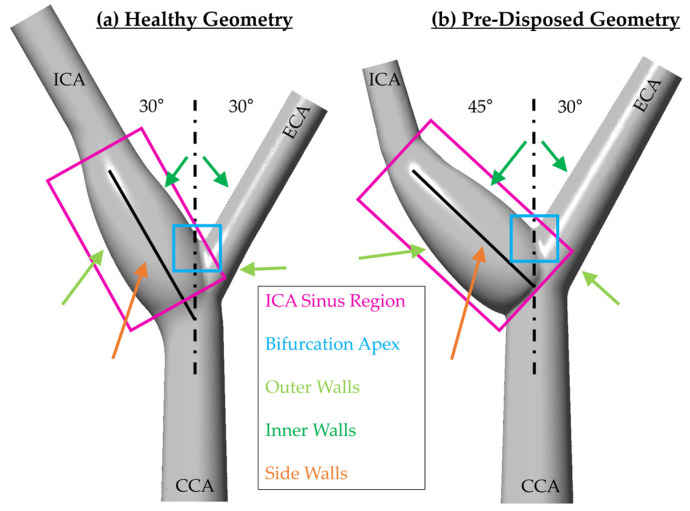
‘Healthy’ (**a**, left) and ‘pre-disposed’ (**b**, right) model geometry of the carotid artery bifurcation: (**a**) symmetric total branching angle with 30° ICA branching angle, characteristic of a ‘healthy’ geometry; (**b**) asymmetric total branching angle with 45° ICA branching angle, characteristic of a ‘pre-disposed’ geometry. Regions of interest are highlighted.

The average person is not affected by clinically relevant carotid stenosis (over 50% severity) as stenoses appear in only about 2–8% of the population [48]). Among others, a factor increasing the atherosclerosis risk is the vessel’s geometry, i.e., its anatomical features. Clinically, two main geometrical risk factors have been identified: (i) larger bifurcation angle, especially larger ICA angles, and (ii) a small ICA/CCA diameter ratio [49,50,51]). To study the influence of these geometrical risk factors, computed flow fields of two carotid artery bifurcation models were compared. The first is based on vessel measurements from healthy volunteers without any specific increased atherosclerosis risk. Our ‘healthy’ geometry model’s dimensions were based on these healthy patient averages [52,53,54]. The CCA had an inlet diameter of 6.5mm and branched into the ICA and ECA with a symmetric 60° angle (Figure 2a). Secondly, a ‘pre-disposed’ geometry was modeled, incorporating both known geometrical risk factors. An asymmetric branching angle was realized by increasing the ICA angle to 45° [49], and the ICA/CCA diameter ratio was reduced to 0.5 by reducing the ICA diameter [34,50] (Figure 2b). The ICA sinus was included in both geometries as it is a strong proatherogenic region. Entry and exit lengths were added to the ICA and ECA to improve numerical convergence, prevent nonphysical reverse flow at the outlet, and achieve a developing inflow.

#### 2.1.1. Healthy Geometry

The modeled ‘healthy’ geometry dimensions are based on the physiological average of a healthy volunteer population [52,53,54]. The geometry had a circular 6.5 mm CCA inlet diameter and the CCA diameter tapered continuously toward the symmetric bifurcation. As it is a crucial region for atherosclerotic plaque formation, the ICA sinus was incorporated in the model geometry according to Figure 2.

#### 2.1.2. Pre-Disposed Geometry

This novel pre-disposed geometry represents a patient cohort with an increased risk for atherosclerotic plaque formation and is based on this patient population average. Based on clinical data, the two main geometrical risk factors clinically known to be associated with increased atherosclerosis risk—(i) an increased ICA angle, resulting in an asymmetric total CAB bifurcation angle, and (ii) a reduced ICA/CCA diameter ratio—were incorporated into this ‘pre-disposed’ geometry. The model is a variant of the healthy geometry, where CCA and ECA specifications remain unchanged. First, the ICA angle was increased to the typical 45° [49], resulting in an asymmetric and larger total bifurcation angle. Secondly, the ICA/CCA diameter ratio was reduced to 0.5 [50] between the inlet and ICA downstream of the sinus by reducing the ICA diameter (Table 1). The development of this pre-disposed geometry allowed us to study how the flow field differs between a healthy and pre-disposed population and how differences in forming vortical structures might influence atherosclerosis, triggering wall shear stress distributions.

### 2.2. Computational Fluid Dynamics (CFD)

Computational fluid dynamics (CFD) was used to compute three-dimensional time-resolved flow fields in both geometries. ANSYS^®^ meshing tool was used to discretize both geometries. ANSYS^®^ FLUENT Academic Research Mechanical Release 2021 R1 was used to specify the physics of the Navier–Stokes equations using a finite volume method and a pressure-based solver under time-dependent laminar conditions. A CFD solver was chosen due to the rigid wall approximation, which allowed for the comparison of three-dimensional flow structures between the two fixed geometries. A high-performance computing cluster (Pegasus, GWU) was used to solve the Navier–Stokes equations. ANSYS^®^ FLUENT 2021 R2 and Python JUPYTER Notebook 6.4.8 were used to post-process the results.

#### 2.2.1. Spatial and Time Discretization

Both domains were discretized using tetrahedral core meshing and boundary layer inflation meshing at the vessel’s lumen wall. A mesh independence study was performed by varying both mesh variables and observing the change in local velocity as well as wall shear stresses over the wall. High accuracy, and thus mesh independence, were achieved and validated, with a total of 2,080,502 mesh elements in the domain. Several cycles of the physiological inflow waveform were run until independence from the initial conditions and cycle-to-cycle repeatability was achieved. Data from the third physiological cycle were analyzed and are reported herein. The fixed time step was set to ensure a maximum Courant–Friedrichs–Lewy (CFL) number of less than 1 for all timesteps.

#### 2.2.2. Boundary Conditions

**Figure 3:**
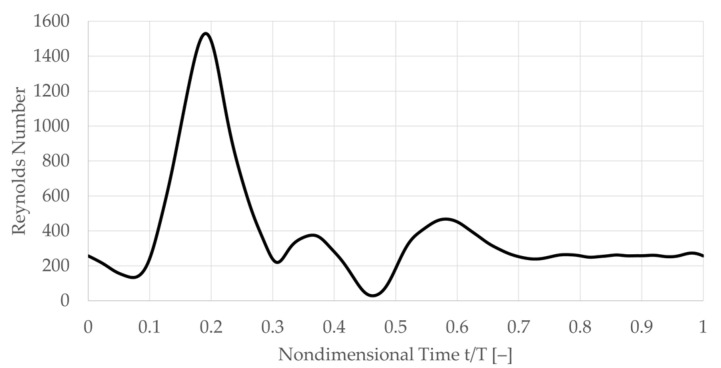
Physiological pulsatile inflow into the common carotid artery inlet over one cardiac cycle. The diagram represents the time dependence of the total inflow velocity magnitude at the inlet boundary. Waveform is the patient-averaged CCA flow of a healthy patient population [56].

The carotid artery bifurcation model was subjected to physiological pulsatile flow, which was provided at the CCA through a time-varying uniform velocity inlet condition. Mass outflow was controlled at the ICA and ECA outlet, and the rigid walls’ no-slip boundary conditions were applied. Medium and large-sized vessels, such as the carotid artery bifurcation, experience negligible non-Newtonian effects [55], so a Newtonian fluid assumption was used. To facilitate interdisciplinary comparison with mechanotransduction-related cell studies, a constant kinematic viscosity of $6.95\cdot 10^{-7}{\;m}^{2}/\sec$ was used. The flow is Reynolds number matched to physiological blood flow in this vessel, where the CCA inflow Reynolds number is defined as CCA inflow velocity, multiplied by the CCA diameter and divided by the kinematic viscosity. The CCA inlet Reynolds number (Re) waveform peaks at Re = 1530, with a mean Re of 385. This inflow waveform is based on patient-averaged flow in the CCA [56] based on a healthy patient cohort and has a physiological period (T) of 1 s, corresponding to a heartbeat frequency of 60 bpm. For the user-defined velocity function at the inlet, a digitized waveform was used. The digitized model was devel-oped in our lab. The original data (which we digitized) is from Holdsworth et al. [57,58,59,60] (Figure 3).

At the ICA and ECA outlets, the mass outflow was controlled with a resistive outlet, implemented through a pressure outlet boundary condition. This resistive outlet condition was chosen due to its numerical robustness and nearly identical behavior to traditional three-element Windkessel models [61]. Resistive outlet conditions allowed for the control of the flow split between the ICA and ECA outlets and matched physiological clinical data. They are defined through a pressure outlet as they follow Δp=R·Q, where Δp is the pressure drop in Pascals, R is the vascular resistance in $\left[\mathrm{Pa}\cdot s/m^{3}\right]$, and Q is the volume flow rate in [$m^{3}/s$]. Using resistive outlet conditions in the CFD simulation, resistance values (R) for both branches were iteratively changed until the mass outflow of the ICA matched patient-averaged clinical data [62]. To ensure continuity, ECA outflow was defined as CCA inflow minus ICA outflow. The values of vascular resistances iteratively determined and used in the ‘healthy’ as well as ‘pre-disposed’ geometry were $R_{\mathrm{ICA}}=1.5\cdot 10^{6}\;\mathrm{Pa}\cdot s\cdot m^{-3}$ and $R_{\mathrm{ECA}}=11\cdot 10^{6}\;\mathrm{Pa}\cdot s\cdot m^{-3}$ for the ICA and ECA, respectively, for both geometries. Thus, the outlet pressure boundary conditions were time-dependent functions due to their linear dependence on the physiological inflow waveform and their respective resistances.

### 2.3. Time-Averaged Wall Shear Stress and Vortex Identification

Wall shear stresses (WSS) are a main driver of atherosclerotic plaque formation, and thus WSS metrics provide an indication of expected cell response and disease onset. By contrast, time-averaged-WSS (TAWSS) is a commonly used metric, and this study focused on instantaneous WSS, as in our prior study, the observed WSS differences in the ‘pre-disposed’ geometry resulted in a proatherogenic TAWSS distribution [63]. Furthermore, this allowed for an increased understanding of the instantaneous flows and stresses underlying an unfavorable TAWSS distribution. WSS and TAWSS are based on Equations (1) and (2), respectively, where μ is the dynamic viscosity, $\vec{u}$ the local flow velocity, and T the period of the cardiac cycle.

(1) $$\mathrm{WSS}=\vec{\tau_{w}}=\mu \cdot {\left(\frac{\partial \vec{u}}{\partial y}\right)}_{wall}$$

(2) $$\mathrm{TAWSS}=\frac{1}{T}\cdot \;\int_{0}^{T}\left|\vec{\tau_{w}}\right|\;\mathrm{dt}$$

Flow structures such as vortices can significantly impact the WSS distribution. Currently, there are only few investigations of vortical structures in bifurcating vessels, specifically the CAB, and rarely are these investigated with respect to their influence on the WSS distribution. The resulting vortical structures arise from secondary flows due to the vessels’ curvature when branching from the CCA into the ICA or ECA, respectively. Secondary flow structures are expected to resemble the features observed in curved pipes under physiological pulsatile inflow, similar to the results reported by Cox et al. [41]. In curved pipe flow, a counter-rotating ‘Dean’ vortex pair is present, which forms in the flow acceleration phase. Around the inflow peak, this counter-rotating vortex pair typically gets deformed, and a second vortex pair, rotating in the opposite direction, will form. During the inflow deceleration phase, a separation or split of the deformed main counter-rotating vortex pair (Dean vortex) can be observed in pipe flow [41]. This study revealed similar secondary flow patterns in the bifurcating vessel as previously observed in a single curved pipe [64,65].

The identification of three-dimensional vortical structures in the flow field was performed using the $\lambda_{2}$ criterion, one of the most accepted vortex identification techniques [66], which is widely used in cardiovascular fluid dynamics. Furthermore, its advantage is that it is a quantitative method as it is based on the physical definition of the vortical structure and does not depend on a visualization technique [67]. The $\lambda_{2}$ criterion is an Eulerian and eigenvalue-based vortex identification method [68]. The three-dimensional velocity field functions as input, where the velocity field is described as $\vec{u}=\left(u,\;v,\;w\right)$. From this, the velocity gradient tensor J is computed using Equation (3).

(3) $$J=\left[\begin{array}{ccc} \frac{\partial u}{\partial x} & \frac{\partial u}{\partial y} & \frac{\partial u}{\partial z}\\ \frac{\partial v}{\partial x} & \frac{\partial v}{\partial y} & \frac{\partial v}{\partial z}\\ \frac{\partial w}{\partial x} & \frac{\partial w}{\partial y} & \frac{\partial w}{\partial z} \end{array}\right]$$

This velocity gradient tensor is decomposed into the strain deformation tensor S (Equation (4)), which is its symmetric part, and the rotation rate tensor Ω (Equation (5)), its asymmetric part [68].

(4) $$S=\frac{J+J^{T}}{2}$$

(5) $$\Omega =\frac{J-J^{T}}{2}$$

The eigenvalues of $\left(S^{2}+\Omega^{2}\right)$ are computed and labeled such that $\lambda_{1}\geq \;\lambda_{2}\geq \;\lambda_{3}$. A voxel is detected to be part of a vortex core if it has two negative eigenvalues; thus, if $\lambda_{2}<0$. The vortex region is defined as a connected region of $\lambda_{2}<0$ voxels [67,69,70,71]. To highlight strong vs. weak vortical structures, an additional threshold of $\lambda_{2}$ can be applied [67]. When analyzing $\lambda_{2}$, we visualized, the three-dimensional iso-surfaces of the chosen $\lambda_{2}$ threshold.

To isolate and track key vortices originating in the wall boundary layer a PYTHON code was developed for post-processing. A vortex core was defined as a local pressure minimum, and thus the exact location of the vortex origin at the wall was identified by tracking a local pressure minimum at the vessel wall. This allowed for the analysis of the moment in time and location for vortex formation and its evolution over the cardiac cycle. The vortical structure of interest was first identified using the $\lambda_{2}$ criterion defining the broad location of vortex origin, e.g., the sinus side wall. To guarantee that the pressure minimum location of the vortex of interest was captured, a three-dimensional reduced field of view or search window was created and kept stationary or moved with the bulk flow velocity downstream where applicable. The location of the vortex core within this search window was identified based on the position of the pressure minimum on the three-dimensional surface in the search window for each time step. The position was provided in a translated and rotated coordinate system, where the origin lay in the bifurcation point and the axial downstream direction followed the ICA sinus center axis (following a black solid line in the sinus, as illustrated in Figure 2). The path traced by the pressure minimum over time was provided for one side of the vessel due to its symmetry around the z-axis. The following results will present the location, velocity, and acceleration of a main vortex core’s origin at the sinus side wall.

## 3. Results

The results show WSS, secondary flow fields, and three-dimensional vortical structures for a typical ‘healthy’ and ‘pre-disposed’ carotid artery bifurcation geometry. The analysis focused on the ICA sinus as it is the prevalent region for atherosclerotic plaque formation in the CAB. The results highlight the impact that large vortical structures have on the WSS distribution, hinting at their crucial role in atherosclerosis. This is followed by the analysis of the development of a main hairpin vortical structure in the ICA sinus, specifically, its occurrence time window within the cardiac cycle and its evolution. The results highlight the importance of three-dimensional vortical structures within the CAB to increase the understanding of hemodynamic effects underlying proatherogenic WSS distributions.

### 3.1. Influence of Three-Dimensional Vortical Structures on WSS

Wall shear stress is widely known to be one of the key stresses affecting endothelial cells and plays a major role in atherosclerosis. Thus, the flow fields within the CAB significantly determine whether a physiological or pathological shear stress distribution emerges. There is a gap in the literature on the study of complex flow structures such as vortices, present in the CAB flow, and how they impact the WSS distribution. In this section, vortical structures and concomitant instantaneous WSS are evaluated to gain deeper insights into the flows related to carotid artery bifurcation.

**Figure 4:**
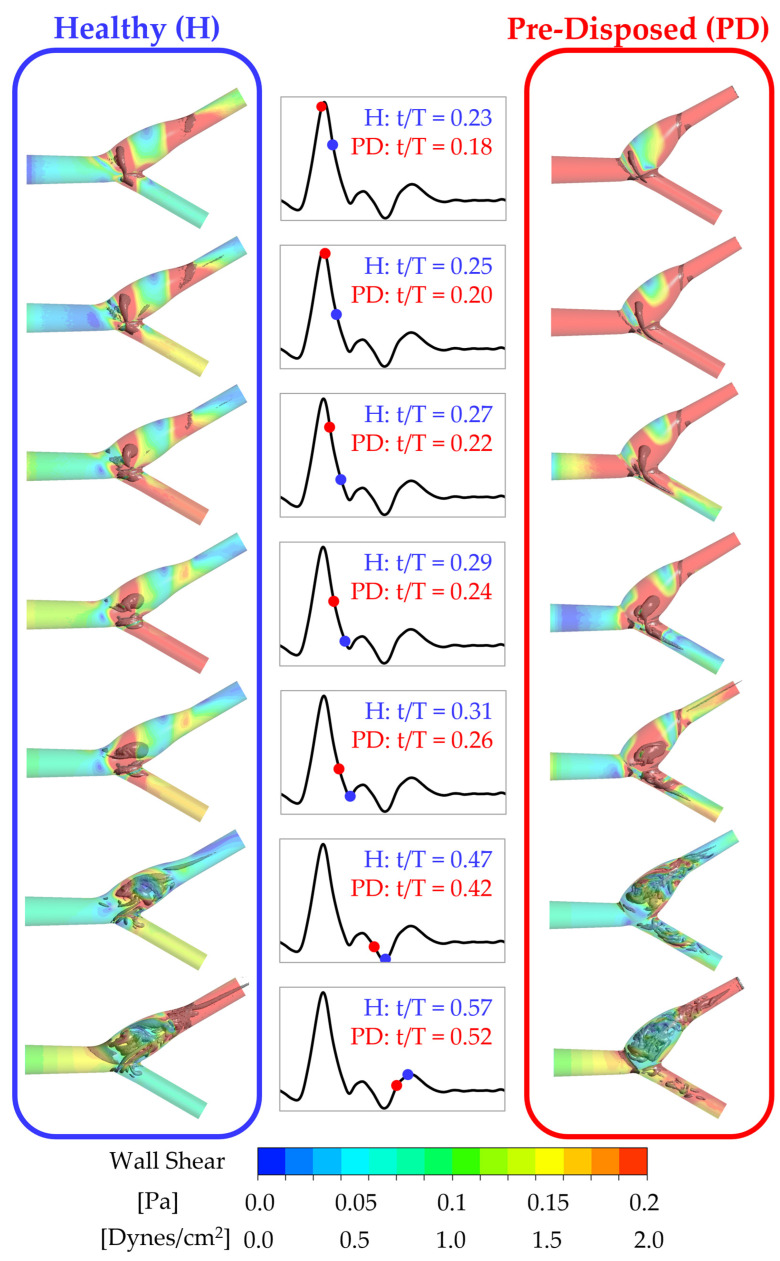
Wall shear stress distribution and the underlying three-dimensional vortices. The wall shear stress distribution is shown as slightly transparent (color bar on the bottom), and vortices in the vessel volume are shown in gray using a $\lambda_{2}$ threshold. The ‘healthy’ geometry is shown in the left column (famed in blue), and the ‘pre-disposed’ geometry is shown in the right column (framed in red). The middle column insets show an instant in the cycle for the ‘healthy’ and ‘pre-disposed’ geometry with a blue and red dot on the pulsatile inflow waveform, respectively.

Three-dimensional vortices are found to strongly influence the wall shear stress distribution. This can be seen in Figure 4, where the ‘healthy’ geometry is shown on the left (framed in blue), and the ‘pre-disposed’ geometry is shown on the right (framed in red). Slightly transparent wall shear stress contours are shown on the CAB walls. Vortices present in the fluid volume are visualized in a gray solid color using the $\lambda_{2}$ criterion. The middle row inset indicates the moment in time of the instantaneous result with dots on the physiological waveform in blue and red for the ‘healthy’ and ‘pre-disposed’, respectively. For both geometries, a strong, hairpin-shaped vortical structure forms in the ICA sinus. This main hairpin vortical structure starts to form at an earlier instance in the cardiac cycle in the case of the ‘pre-disposed’ geometry and then for the ‘healthy’ geometry.

Starting with the ‘healthy’ geometry (left, in blue), it is observed that this hairpin vortical structure changes its orientation over the cardiac cycle. The vortical structure starts forming in the inflow deceleration phase (t/T = 0.23) at an upstream sinus position around the CAB branching point. Initially, the vortical structure is oriented in extension to the CCA, so its imaginary center axis is almost parallel to the CCA center axis. By the end of the inflow deceleration phase (t/T = 0.31), the main hairpin vortical structure’s center axis is almost perpendicular to the ICA center axis. Thus, throughout the inflow deceleration, this main hairpin vortical structure rotates, orienting its ‘head’ in the axial ICA flow direction. The ‘pre-disposed’ geometry (right, framed red) shows a similar phenomenon, forming one main hairpin vortical structure that changes its orientation in a similar pattern. The formation of this main hairpin vortical structure starts earlier in the cardiac cycle than in the ‘healthy’ case. In addition, by comparing the vortical structure’s orientation over time, a time shift in the vortical structure’s orientation can be observed, t/T=0.05 earlier means: 5% of the total cardiac cycle length earlier AS WELL AS 13% earlier relative to the total at-tached lifespan of the ‘pre-disposed’ hairpin vortical structure. Previous studies have established that wall shear stress is an essential mechanism that drives the biochemical mechanotransduction process related to atherosclerotic plaque formation [8,10,11]. Typically, TAWSS is studied to capture effects over the entire cardiac cycle, and it is known that low TAWSS are proatherogenic. Herein, the instantaneous time-resolved WSS results show that, throughout the cardiac cycle, various locations of the ICA sinus experience low WSS for both geometries. Both geometries, however, show a high-WSS band across the sinus, and furthermore, the position of this high-WSS band aligns with the internal position of the main hairpin vortical structure. This clearly shows the strong influence of vortical structures on the wall shear stress distribution. From these results, we anticipate a positive physiological impact of this hairpin vortical structure as it increases WSS in the generally low-WSS flow separation zone at the ICA sinus side and outer walls. This vortical structure’s behavior will be discussed in more detail in Section 3.3.1. Thus, this study increases current knowledge by determining vortical structures as a hypothesized cause for a proatherogenic wall shear stress distribution.

Thus, in summary, both geometries, ‘healthy’ and ‘pre-disposed’, develop a main hairpin vortical structure in the ICA sinus. These results demonstrate the strong influence of this observed hairpin vortical structure on the WSS distribution. Its internal location is linked to an increased WSS in an otherwise low-WSS separation zone. Thus, this hairpin vortical structure could have a positive physiological impact by increasing WSS to nonatherogenic values in the critical sinus region. Therefore, it is important to deepen our knowledge of vortical structure formation in the CAB to understand its possibly crucial role in atherosclerosis and plaque formation.

### 3.2. Secondary Flows in the ICA Sinus

The CAB’s branching results in a curvature within the vessel’s axial direction and thus secondary flows are presented. The previous section showed the impact of three-dimensional vortices on WSS. As secondary flows facilitate vortical structure formation, the evolution of secondary flows over the cardiac cycle is investigated and compared between a ‘healthy’ and a ‘pre-disposed’ geometry in this section. The focus again lies on the ICA sinus as it is the location mostly affected by atherosclerosis.

**Figure 5:**
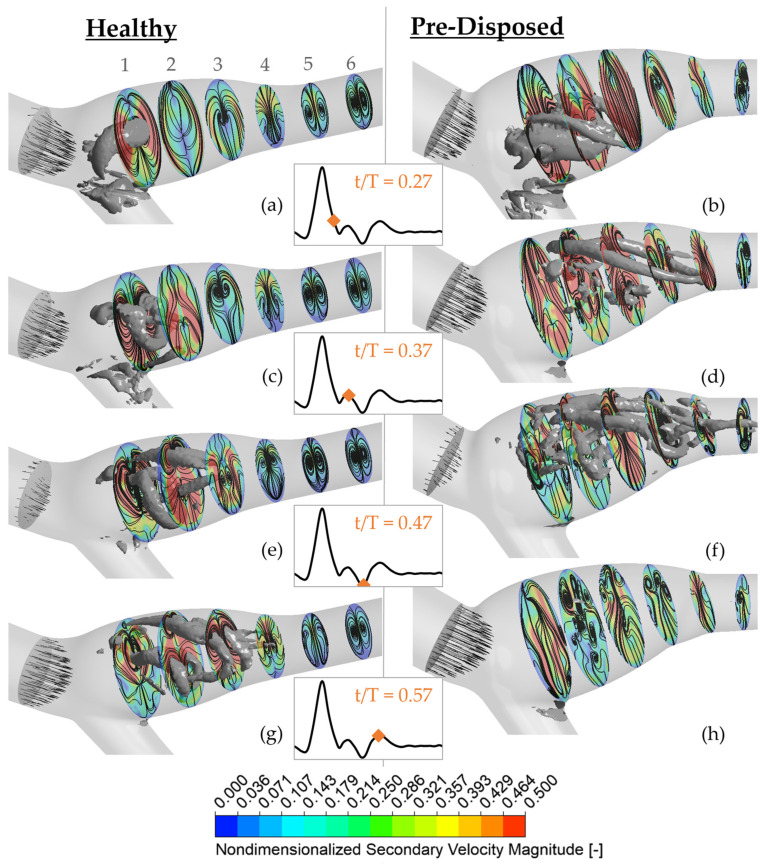
Secondary velocities in the ICA sinus over the cardiac cycle. ‘Healthy’ geometry is on the left (**a**,**c**,**e**,**g**), and ‘pre-disposed’ geometry is on the right (**b**,**d**,**f**,**h**). The middle insets show the analyzed instant in time, which is highlighted with an orange diamond on the pulsatile inflow waveform. The axial inflow in the CAB just upstream of the bifurcation is indicated with black axial velocity vectors. Velocity results are presented on 6 planes perpendicular to the ICA center axis; planes are labeled 1-6 where 1 is most upstream near the CCA and 6 is the most downstream (labeled in **a**). Nondimensionalized secondary velocity magnitude (u/U) (secondary velocity magnitude divided by U = U (t/T = 0.27), where U is the mean velocity magnitude at the CCA inlet) is defined with the color bar, and streamlines are added in black. Three-dimensional vortical structures are determined using a constant $\lambda_{2}$ criterion over all time instances t/T and shown in gray.

The secondary velocity magnitude is shown at six planes perpendicular to the ICA branching centerline path (color bar) (see Figure 5). Planes are labeled 1–6, with 1 being most upstream, close to the CCA branching point, and 6 most downstream closer to the ICA outlet (as labeled in Figure 5a). The secondary velocity streamlines are added in black over the magnitude contours, and the instantaneous axial bulk inflow is represented with the black axial vectors in the CCA. Three-dimensional vortical structures using a $\lambda_{2}$ threshold are shown in gray. The ‘healthy’ geometry is shown on the left (a), and the ‘pre-disposed’ geometry is presented on the right (b). The time instances of data are indicated with the orange diamonds on the pulsatile inflow waveform in the middle inset.

Toward the end of the inflow deceleration phase at t/T = 0.27 (Figure 5a,b), both geometries exhibit a pronounced hairpin vortical structure in the ICA sinus. In the ‘healthy’ geometry (Figure 5a), it is located around plane 1. The vortical structure’s intersection with the imaginary plane can be seen in the curved high-density streamlines at its location. Thus, a counter-rotating vortex pair is visible in plane 1. This location also exhibits a high secondary velocity magnitude that is concentrated at the sinus side walls. The secondary velocity magnitude weakens downstream, where no three-dimensional vortical structures with comparable strength are present. Similarly, counter-rotating vortex pairs can be observed further downstream, in planes 5 and 6. The ‘pre-disposed’ geometry generally experiences much higher secondary velocities in that phase (Figure 5b). At plane 1, a similar counter-rotating vortex pair is observed where the three-dimensional vortical structure intersects. This vortex pair is located closer to the inner sinus wall and can similarly be found in plane 2. In contrast to the ‘healthy’ geometry, no clear symmetric counter-rotating vortex pairs can be observed in planes 5 and 6. Thus, the vortical structure remains more coherent and persists further downstream in the healthy geometry.

Shortly after the strong inflow deceleration phase at t/T = 0.37, the three-dimensional hairpin vortices experience significant changes in both geometries. In the ‘healthy’ geometry very dense streamlines and high secondary velocity magnitudes around the vortical structure legs are observed, where its head turns toward the inner sinus wall (Figure 5c). The highest secondary velocities are still concentrated in the first upstream half of the ICA sinus. In contrast, high secondary velocities occupy the full sinus region in the ‘pre-disposed’ geometry (Figure 5d). Circular streamlines clearly indicate the location of three-dimensional vortices.

At the inflow minimum at t/T = 0.47, high secondary velocity is still concentrated in the sinus region, mostly in the upstream regions on planes 1 and 2 for the ‘healthy’ geometry (Figure 5e). Circular streamlines in plane 3 indicate that the observed bonelike vortical structure passes through the hairpin vortical structure loop, and plane 2 continues at lower $\lambda_{2}$ values downstream. Very dense circular streamlines are visible in all planes, indicating the presence of long ‘leg-like’ three-dimensional vortical structures through the whole ICA. The ‘pre-disposed’ geometry has very different secondary flow patterns (Figure 5f). Here, higher secondary velocities are present in the downstream half of the sinus. Similar to the ‘healthy’ geometry, three-dimensional structures and counter-rotating vortex pairs are mostly located close to the sinus outer wall. But the upstream sinus part (around planes 1 and 2) contains many small, very disordered vortical structures, disorganized streamlines, and only small disconnected areas of high secondary velocity.

Finally, secondary flows and vortical structures exhibit significantly different behavior between the two geometries at t/T = 0.57, when the inflow starts to level off in the ‘diastolic’ phase. In the ‘healthy’ geometry (Figure 5g), one clear counter-rotating vortex pair is identifiable in each plane, and the large three-dimensional structures are still connected and mainly concentrated in the sinus. Beyond the sinus, the flow keeps its organized structure of one clearly defined vortex pair (see planes 5 and 6) throughout the complete time range. For the ‘pre-disposed’ geometry, no three-dimensional structures of comparable strength exist in this late stage in the cardiac cycle (Figure 5h). The secondary velocity magnitude is generally low with only small disconnected local increased secondary velocities. No clear counter-rotating vortex pairs are observed in the ‘pre-disposed’ geometry, neither downstream of the sinus (plane 6) nor in the sinus. Furthermore, when analyzing streamlines, a very disordered secondary flow can be observed, especially mid-sinus as is visible by many small circular streamline islands in planes 2–4 located toward the outer sinus wall.

In summary, a difference in the secondary flow magnitude over the full cardiac cycle is observed between the two geometries. The ‘pre-disposed’ geometry experiences larger regions of strong secondary flows in the inflow deceleration phase and smaller regions in the ‘diastolic’ phase compared with the ‘healthy’ geometry. The ‘healthy’ geometry exhibits a distinct counter-rotating vortex pair throughout the cardiac cycle—as expected for a curved vessel. The ‘pre-disposed’ geometry, on the other hand, lacks those characteristic counter-rotating vortex pairs throughout most of the vessel over most of the cycle. The ‘pre-disposed’ geometry has disturbed flow, with small disordered vortices and small disconnected areas of high and low secondary flow velocities.

### 3.3. Characterization of the ICA Sinus Vortical Structure

The three-dimensional structures significantly influence the shear stress distributions, especially in the strongly affected ICA sinus area. Thus, this section describes vortical structure formation and evolution throughout the cardiac cycle in the ICA sinus. It focuses on the main hairpin vortical structure due to its high impact on the wall shear stress. This analysis aims to explain the flow characteristics underlying a pathological proatherogenic WSS distribution for the ‘pre-disposed’ geometry versus a physiological WSS distribution for the ‘healthy’ geometry. An increased understanding of the role of vortices’ association with atherosclerosis has the potential to become an important tool in early detection, treatment, and intervention planning.

#### 3.3.1. Instant of Formation, Duration, and Rotation of Sinus Vortical Structure

**Figure 6:**
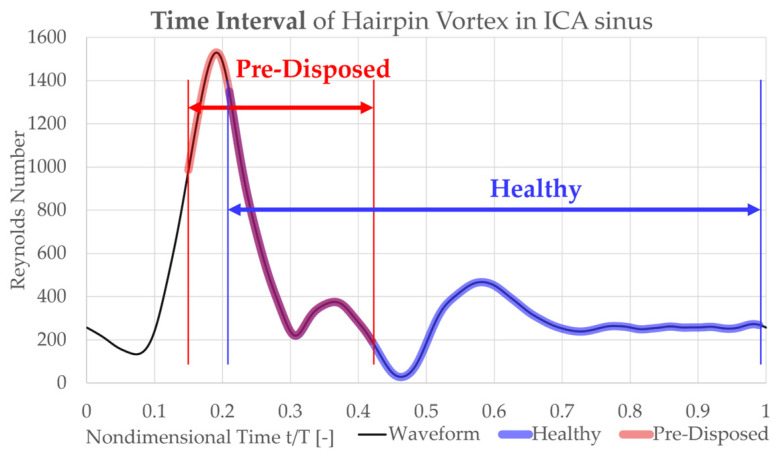
The life span (wall attachment) of the main hairpin vortical structure is shown on the pulsatile inflow waveform (black) for the ‘healthy’ geometry in blue and for the ‘pre-disposed’ geometry in red. The hairpin vortical structure is observed earlier for the ‘pre-disposed’ case and also stops to be detected significantly earlier.

The occurrence and orientation of the main hairpin vortical structure in the ICA sinus (described in Section 3.1) were investigated further. While this pronounced hairpin vortical structure forms in both cases, the temporal starting point of its formation and its duration vary between the cases. The hairpin vortical structure is visualized by choosing a $\lambda_{2}$ threshold to filter out vortices of smaller scale and strength. The existence window of the hairpin vortical structure is defined as its presence using this constant threshold. This lifespan of the vortical structure is plotted in Figure 6 over the pulsatile inflow waveform in blue and red for the ‘healthy’ and ‘pre-disposed’ cases, respectively. Several differences are evident. As was found from the three-dimensional vortical structure contours analyzed in Section 3.1, the formation of the main hairpin vortical structure starts earlier for the ‘pre-disposed’ geometry (t/T = 0.05 earlier). Notably, its formation starts in the inflow acceleration phase, whereas for the ‘healthy’ geometry, the hairpin vortical structure only starts to develop shortly after the inflow peak, in the inflow deceleration phase. In the ‘pre-disposed’ geometry, the hairpin vortical structure loses its dominance compared with the other vorticity in the sinus, where this endpoint of its predominance is observed at t/T = 0.42, shortly after the main inflow deceleration. The hairpin vortical structure in the ‘healthy’ geometry persists much longer than for the ‘pre-disposed’ case, almost to the end of the cardiac cycle (t/T = 0.98). These results highlight two main differences between the distinct hairpin vortical structure in the ICA sinus. In a ‘healthy’ geometry, the vortical structure starts forming only in the inflow deceleration and persists almost three times as long as that in ‘pre-disposed’ geometry.

#### 3.3.2. Axial Pressure Gradient over ICA Sinus

**Figure 7:**
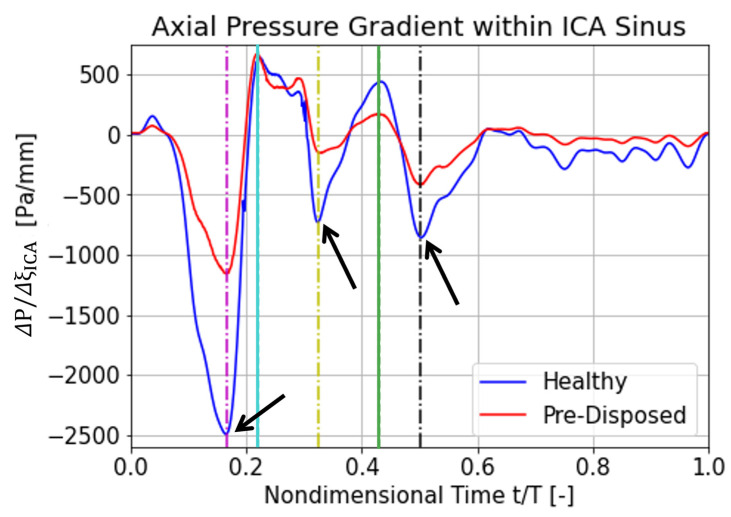
Axial pressure gradient over the ICA sinus through the cardiac cycle. ‘Healthy’ geometry is in blue, and ‘pre-disposed’ is illustrated in red. The indicated moments in time are shown with dashed lines: t/T = 0.165 (magenta), t/T = 0.22 (cyan), t/T = 0.325 (yellow), t/T = 0.43 (green), and t/T = 0.50 (black). Lines indicating favorable pressure gradients are marked with dash dots, and lines indicating adverse pressure gradients are solid. Large differences in the favorable pressure gradient are observed, which are higher for the ‘healthy’ geometry (highlighted with the arrows). Smaller differences are evident in the case of an adverse pressure gradient.

The following section focuses on explaining a possible cause for the different hairpin vortical structure characteristics between a ‘healthy’ and a ‘pre-disposed’ geometry analyzed before. We argue that the differences in the axial pressure gradient over the ICA sinus—caused by its differing geometrical resistance—might cause discrepancies in vortical structure behavior. A favorable flow-driving pressure gradient is defined to be negative ($\Delta P/\Delta \xi_{ICA}<0$), whereas an adverse pressure gradient is positive ($\Delta P/\Delta \xi_{ICA}>0$). The axial direction is defined as the direction of the ICA center axis. For the presented analysis, $\Delta \xi_{ICA}$ spans over the length of the ICA sinus. The results for the axial ICA pressure gradient over the cardiac cycle are shown in Figure 7, for the ‘healthy’ and ‘pre-disposed’ geometry in blue and red, respectively. Local pressure gradient minima and maxima are indicated by the lines, where the magenta, yellow, and black lines highlight favorable pressure gradient extrema, and the cyan and green lines indicate adverse pressure gradient extrema.

Significantly large differences are observed for the favorable pressure gradient extrema. The first flow-driving pressure gradient is the strongest pressure gradient over the entire cardiac cycle, experiences a very steep slope, and has the largest peak difference between the geometries (Figure 7, magenta line). The pre-disposed geometry experiences a 55% lower pressure gradient at this first peak. The maximum adverse pressure gradient is very similar for the two geometries (Figure 7, cyan line). The ‘pre-disposed’ geometry experiences an almost equal adverse pressure gradient to the ‘healthy’ geometry in the first peak (cyan line). Progressing in time, the ‘healthy’ geometry experiences another strong, favorable pressure gradient (yellow line); this pressure gradient peak is about 75% lower for the pre-disposed geometry. In the later stages of the cardiac cycle, the hairpin vortical structure is detected only for the ‘healthy’ geometry. As indicated by the green line (Figure 7), the ‘healthy’ geometry again experiences a stronger adverse pressure gradient. The last significant favorable pressure peak, before the pressure gradient levels off at a low value for both geometries, is located around t/T = 0.5, indicated by the black dashed line in Figure 7.

In summary, the adverse axial pressure gradient time trace within the ICA sinus has a very similar shape for the two geometries, especially in the time window where a vortical structure is detected for both. Large differences are, however, observed in the favorable pressure gradient magnitude, where the ‘healthy’ geometry experiences much stronger driving pressure gradients. These low favorable pressure gradients in a pre-disposed geometry are hypothesized to promote a proatherogenic vortical structure formation, resulting in a pathological wall shear stress distribution.

#### 3.3.3. Streamwise Motion of Vortex Core

In this section, the streamwise motion of the main hairpin vortical structure is presented. The investigation of its motion characterizes the main hairpin vortical structure and identifies the differences between a ‘healthy’ and ‘pre-disposed’ geometry. Furthermore, it provides insights into the location and motion of the high-WSS band in the sinus.

**Figure 8:**
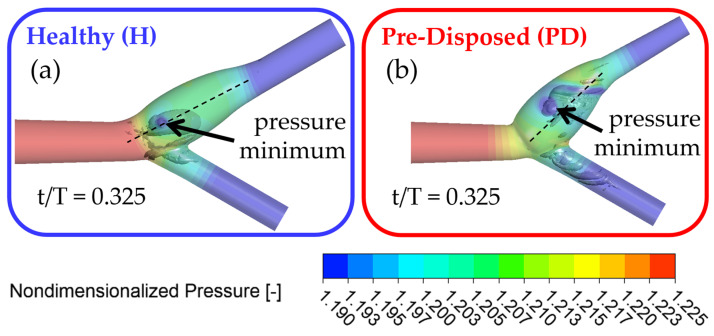
Nondimensionalized static pressure on artery wall (transparent, color bar) with underlying three-dimensional vortical structure using $\lambda_{2}$ vortex identification. Pressure is nondimensionalized by dividing by P0, where P0 = P(t/T = 0.191) at the CCA inlet center for the entire cardiac cycle. The illustrated instantaneous moment is t/T = 0.325. The pressure minimum on the sinus side wall shows the position of the vortical structure core of the main hairpin vortical structure: (**a**) blue shows the ‘healthy’ geometry; (**b**) red indicates the ‘pre-disposed’ geometry.

As previously observed, the vortical structure forms around the bifurcation point and tilts into the ICA. In what follows, ‘axial motion’ is used to describe the vortices’ motion along the ICA axial flow direction in the translated and rotated coordinate system, as indicated in Section 2.3. Figure 8 shows the three-dimensional vortical structures in the $\lambda_{2}$ criterion in gray in the vessel and the nondimensional pressure is shown with slightly transparent wall contours. Both geometries are shown after the inflow deceleration phase at t/T = 0.325, with the ‘healthy’ geometry framed in blue (Figure 8a) and the ‘pre-disposed’ geometry framed in red (Figure 8b), respectively. The core of a three-dimensional vortical structure is defined with a pressure minimum condition. Pressure contour plots allow for the visualization of the pressure origin. It is evident that the main hairpin vortical structure originates from the ICA sinus side walls, as indicated by the local pressure minimum on the sinus walls for both geometries (Figure 8) that matches the underlying hairpin vortical structure. In the following section, the motion of the pressure minimum at the vortical structure origin on the ICA sinus side wall will be tracked to indicate the position and motion of the hairpin vortical structure.

**Figure 9:**
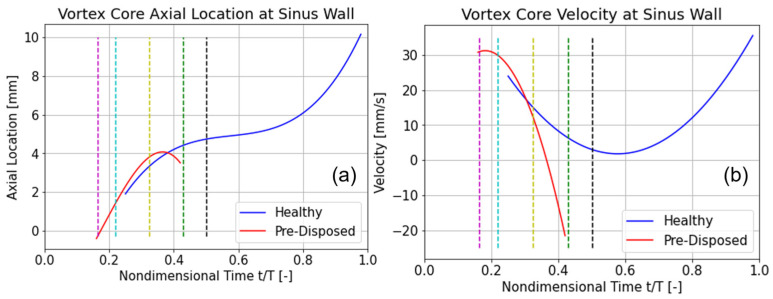
Vortical structure motion over the cardiac cycle. ‘Healthy’ geometry is in blue, and ‘pre-disposed’ is shown in red: (**a**) location of vortex core along axial ICA direction; (**b**) velocity of vortex core. The indicated moments in time are shown with the dashed lines: t/T = 0.165 (magenta), t/T = 0.22 (cyan), t/T = 0.325 (yellow), t/T = 0.43 (green), and t/T = 0.50 (black).

The position of the vortical structure origin was determined by finding the axial location of the pressure minimum in the vortical structure core. The axial position of the vortical structure and velocity are plotted in Figure 9a,b, respectively, over the cardiac cycle, where the ’healthy’ geometry is displayed in blue, and the ‘pre-disposed’ geometry in red. In what follows, the ‘vortex structure core’ will be used to describe the pressure minimum at the vortical structure’s wall origin. Instances of time representing axial pressure minima acting over the vortex core (as discussed in Section 3.3.2) are highlighted with dashed lines and will be elucidated in this and the following section.

The location of the vortex core minimum pressure is plotted in Figure 9a using a translated and rotated coordinate system with the origin at the CAB bifurcation point and streamwise direction aligned with the ICA center axis (as shown in Figure 2 and detailed in Section 2.3). The distinct time shift of vortex pressure minima at the wall can be confirmed. The much earlier occurrence of this wall pressure minimum is clear in the case of the ‘pre-disposed’ geometry, as well as its significantly shorter duration of persistence. For both geometries, the vortex core translates downstream initially. Approximately t/T = 0.2 after onset, the downstream translation (or convection) of the vortex core slows significantly for both geometries. Afterward, a relatively stationary period between t/T = 0.5 and 0.7, the vortex core of the ‘healthy’ geometry again translates downstream at a much higher rate. In the ‘pre-disposed’ geometry, however, the vortex core does not recover from the slowed-down downstream motion, translates upstream very shortly, and then quickly deteriorates completely.

The changes in the downstream motion are further elucidated in the velocity plots (Figure 9b). It can be observed that the velocity of the vortex core initially decreases for both geometries. In the ‘healthy’ geometry, the velocity decreases slowly and nearly reaches a standstill. This very low vortex core translation velocity (‘almost stillstand’) occurs around t/T = 0.6. The vortex core does not completely slow and increases its velocity again, resulting in faster downstream motion. In the case of the ‘pre-disposed’ geometry, the velocity of the vortex core decreases at a nearly constant rate. In this case, the vortex core reaches a stillstand (velocity = 0 mm/s), at which time it is located at the most downstream location (the maximum axial location). The vortex core reaches a negative velocity early after formation, which continues to decrease almost linearly. This results in an upstream vortex core motion, soon followed by its deterioration.

In summary, the hairpin vortical structure initially translates downstream, for both geometries. Both cases then experience a slowed downstream motion, but the vortical structure in the ‘healthy’ case recovers around mid-cycle, avoids stagnation, and continues its downstream motion at a rapid rate. In the ‘pre-disposed’ geometry, on the other hand, the vortical structure starts decelerating early, stagnates, moves upstream, and deteriorates shortly after.

The axial pressure gradient peaks (as described in Section 3.3.2) acting on the hairpin vortical structure are hypothesized to be the driving factor of the vortical structure translation phenomena. Thus, the vortex core behavior will be linked to the acting axial pressure gradient in what follows. The first favorable pressure gradient peak (Figure 7, magenta line) occurs approximately at the time when both hairpin vortices start to form and intersect very closely with the starting point of the vortical structure in the ‘pre-disposed’ geometry (compare the magenta line in Figure 7 and Figure 9a). The ‘healthy’ geometry experiences a 200% stronger initial favorable pressure gradient than the ‘pre-disposed’ geometry.

For the ‘healthy’ geometry, the first adverse pressure gradient peak occurs shortly before the hairpin vortical structure starts to form (compare the cyan line in Figure 7 and Figure 9a). In contrast to the ‘healthy’ geometry, the ‘pre-disposed’ geometry has two distinct peaks in this adverse pressure gradient period, where the second one lies between the cyan and yellow lines in Figure 7. Comparing the second adverse pressure gradient peak (Figure 7, green line) to the occurrences in the vortex core’s motion (Figure 9a,b, green dashed line), we find that it correlates with its slowed downstream position and translation (Figure 9a). In the ‘healthy’ geometry, the final favorable pressure peak (Figure 7, black dashed line) shows an interesting possible effect on the hairpin vortical structure characteristics. The moment of this axial pressure gradient peak is indicated by the black dashed line in Figure 9a,b. This is possibly preventing the vortical structure’s stillstand (Figure 9b) and slowly accelerating the vortical structure’s downstream motion again (Figure 9a,b).

In summary, these results strongly support the hypothesis that the differences in the axial pressure gradient significantly affect vortical structure evolution in the sinus. A favorable pressure gradient has a greater impact than an adverse pressure gradient on the main hairpin vortical structure’s evolution.

## 4. Discussion

This study presents a novel analysis of complex flows, including three-dimensional vortical structures, and wall shear stress distribution in the artery model. In addition to this analysis, the study includes a novel patient-averaged ‘pre-disposed’ CAB geometry, representative of a patient cohort that is clinically at higher risk for CAB atherosclerotic plaque formation but has not yet developed stenosis. The comparison of this ‘pre-disposed’ geometry with a ‘healthy’ geometry elucidates how certain vortical structures promote a proatherogenic WSS distribution. A dominant vortical structure influencing the ICA sinus WSS was identified and characterized over the entire cardiac cycle. This expands the state-of-the-art research, which observed three-dimensional flow structures in carotid artery bifurcation [26,30,31,42] by exploring their dependence on geometrical risk factors and their atheroprotective impact on the wall shear stress distribution.

It was found that the presence of a vortical structure strongly affects the wall shear stress distribution; specifically, a highly influential hairpin vortex was found in the ICA sinus. The ICA sinus is the CAB location most commonly affected by atherosclerosis through typically very low WSS in the sinus flow separation zone [19,30,31]. Interestingly, the hairpin vortical structure increases the WSS significantly. Thus, this finding suggests that the main sinus hairpin vortical structure has a positive physiological impact by increasing ICA sinus WSS to atheroprotective levels.

Secondary flows facilitate vortical structure formation [30,35,37], and significant differences between ‘healthy’ and ‘pre-disposed’ secondary flow fields were detected, related to the observed differences in the vortical structure. During the vortical structure characterization, the main hairpin vortical structure in the ‘pre-disposed’ geometry forms earlier and deteriorates significantly earlier than in the ‘healthy’ case. Similarly, the ‘pre-disposed’ geometry has high secondary velocities earlier in the cardiac cycle but lower secondary velocities in the ‘diastolic’ phase of the cycle in which its hairpin vortical structure deteriorates. Furthermore, the ‘pre-disposed’ case contains more small distributed vortices in the ‘diastolic’ phase in the ICA sinus and does not contain the classical counter-rotating vortex pair expected for a curved vessel. The earlier transition into a disturbed secondary flow and vortical structures in the sinus coincides with the reduced peaks of favorable axial pressure gradients in the ICA. Furthermore, the hairpin vortical structure in the ‘healthy’ geometry, experiencing a higher driving favorable pressure gradient, never slows to zero velocity in its downstream translation, whereas it does under the lower favorable pressure gradient for the ‘pre-disposed’ case. This standstill and the subsequent upstream motion are shortly followed by the deterioration of the vortical structure in the ‘pre-disposed’ case. This strongly reduced favorable pressure gradient might have a strong influence on the vortical structure behavior, slow it down, and result in its deterioration, ultimately causing proatherogenic wall shear stress distributions. The differences present in the ‘pre-disposed’ geometry reduce favorable pressure gradients in the ICA sinus, which is the hypothesized driving force for a long-lasting stable vortical structure. We propose that the downstream motion of a persistent hairpin vortical structure, which increases WSS, is physiologically beneficial. We relate the lower pathological WSS found in the ICA sinus of a ‘pre-disposed’ geometry to weaker distorted secondary flows, mitigating the persistence and stability of the hairpin vortical structure caused by a weaker favorable axial pressure gradient.

The findings support our hypothesis that three-dimensional vortical structures impact WSS distribution, and their premature dissolution might play a significant role in the development of atherosclerotic plaques. This is further supported by the observed differences in vortical structures between a ‘healthy’ and a ‘pre-disposed’ geometry, where the latter experiences a proatherogenic WSS distribution.

### Limitations

Clinically reported patient-averaged geometric anatomical features were used to develop the model vessels, in order to reduce complexity and to account for inter-patient variability. While this allowed us to elucidate the significant impact of three-dimensional vortical structures on a proatherogenic wall shear stress distribution, a further expansion of our study will allow us to augment the findings. Thus, it will be valuable to expand the study to larger datasets of model geometries, allowing for a large parametric study, and thus further investigate a variety of healthy and pathological patient-specific geometries. Future work will also deepen the investigation into the characterization of the main hairpin vortical structure and its behavior under different pressure gradients corresponding to the axial pressure gradient within the ICA sinus. A prospective goal will be to define the clear structural and time-evolution parameter differences of this vortical structure to allow for a clear distinction between a physiological and a pathological CAB vortical structure formation, thus informing the clinical early identification of affected patients through medical imaging in the future.

## 5. Conclusions

Physiological flow in carotid artery bifurcation models was studied to increase the understanding of how vortical flow structures may impose an atheroprotective wall shear stress distribution. The CFD simulations of a ‘healthy’ and a ‘pre-disposed’ CAB model geometry revealed substantial differences in the behavior of the main three-dimensional vortical structures in the ICA sinus. This study revealed a strong correlation between a long-lasting, stably persisting main vortical structure and the related increased WSS, known to have a positive physiological impact on the endothelium. This supports our hypothesis that the formation and persistence of a vortical structure is physiologically beneficial and that the deterioration of this structure would play a significant role in the development of atherosclerosis. The difference in vortical structure behavior not only increases our understanding of why certain vessel geometries are more prone to develop atherosclerosis but also could be observable in medical imaging, leading to a potential clinical diagnostic. The characterization of the main ICA sinus vortical structure is a precursor to developing a promising clinical indicator for atherosclerosis risk. Thus, the advances made in this study could be translated to inform the clinical early detection of patients at higher risk for carotid bifurcation atherosclerosis in the future.

## Figures and Tables

**Table 1:** Main anatomical risk factors in CAB showing geometrical differences between ‘healthy’ and ‘pre-disposed’ model geometry.

Geometrical Risk Factors	‘Healthy’ Model	‘Pre-Disposed’ Model
Total CAB Branching Angle	60°	75°
ICA Branching Angle	30°	45°
Diameter Ratio ICA/CCA ^1^	0.7	0.5

^1^ CCA diameter measured at the inlet; ICA diameter measured downstream of the sinus.

## Data Availability

Data are available upon request due to restrictions. The data presented in this study are available upon request from the corresponding author. The data are not publicly available due to the use of this data in ongoing research and planned future publication by this research group.
